# Inadequate dietary diversity practices and associated factors among pregnant adolescents in the West Arsi Zone, Central Ethiopia: a community-based cross-sectional study

**DOI:** 10.1038/s41598-024-53467-5

**Published:** 2024-02-04

**Authors:** Adane Tesfaye, Mulusew Gerbaba, Dessalegn Tamiru, Tefera Belachew

**Affiliations:** 1 Department of Nutrition and Dietetics, Faculty of Public Health, Institute of Health, Jimma University, Jimma, Ethiopia; 2 Department of Nutrition, School of Public Health, College of Medicine and Health Sciences, Dilla University, Dilla, Ethiopia

**Keywords:** Nutrition, Public health

## Abstract

The aftermath of dietary modifications made during pregnancy has the most substantial effects on nutritional status and birth results, despite the important influence of nutritional reserves. Numerous studies have been conducted on dietary practices and their determinants among pregnant women; however, there is a gap in evidence among pregnant adolescents. Therefore, this study sought to close this gap by examining dietary practices and associated factors among pregnant adolescents in the West Arsi Zone, Central Ethiopia. This community-based cross-sectional study was conducted among 459 pregnant adolescents between February and March 2023. Cluster sampling was used for selecting pregnant adolescents. Structured questionnaires were used for data collection. The data were entered into the Kobo toolbox and exported to SPSS version 25 software for analysis. Dietary diversity was assessed using the 24-h dietary recall method. Binary and multivariable logistic regression analyses were used to identify independent predictors of dietary practices. Odds ratios (ORs) with 95% confidence intervals (CIs) were estimated to identify the factors associated with the outcome variables. A p value ≤ 0.05 indicated statistical significance. The prevalence of inadequate dietary practices among the pregnant adolescents was 78.4% (95% CI 74.3%, 82.8%), and a level of nutritional knowledge [AOR = 2.4, 95% CI (1.82–4.74]; an unfavorable attitude toward dietary diversity [AOR = 4.3, 95% CI 2.9–5.83]; a food insecurity status [AOR = 8.7, 95% CI 2.37–10.24]; and a low perceived severity of poor dietary practices [AOR = 4.7, 95% CI 3.26–5.47]. These factors were significantly associated with inadequate dietary practices among pregnant adolescents. The most frequently consumed foods were starchy foods (81.3%) and pulses (79%), and the least consumed foods were meat (2.8%) and fruits (3.48%). The magnitude of inadequate dietary practices was high, and it was significantly associated with educational, behavioral, and economic status. Nutritional interventions focused on communicating nutritional behavioral changes and strengthening sustainable income-generating strategies are recommended to improve the dietary practices of pregnant adolescents.

## Introduction

It is crucial to consume a variety of nutrients, minerals, vitamins and an adequate amount of energy-rich food to protect mothers’ and developing fetuses’ health during the course of pregnancy and beyond^1,2^. Healthy pregnancies and birth outcomes have been recognized as requiring adequate and balanced nutrition during gestation, which is substantially influenced by nutritional literacy and practice^3,4^.

Preterm birth, low birth weight, and the need for newborn critical care are among the consequences that pregnant adolescents experience more frequently than pregnant women^5,6^. Additionally, there was a strong correlation between teenage pregnancy and higher rates of perinatal mortality, neonatal mortality, child mortality, and stillbirth. The negative effects of teenage pregnancy include long-term morbidity and mortality risks, which are exacerbated by poor dietary intake and malnutrition^7,8^.

Pregnancies during adolescence account for 23% of the burden of disease arising from pregnancy and childbirth, although they represent only 11% of all births worldwide^9^. The growth and development of pregnant adolescents and/or fetuses may be impaired due to high competition for nutrients between the still-growing adolescent and her fast-increasing fetus, commonly known as "nutrient partitioning," and gynecological immaturity compromises optimal fetal growth^10^. According to a study in Ethiopia,^11^ adolescent girls are more vulnerable to undernutrition than adults due to intrahousehold food allocation or lack of decision-making power; moreover, most adolescents are unemployed or minors working, which prevents them from accessing diversified diets^12^. Adolescents’ behaviors (e.g., erratic eating behavior) can also impact their dietary diversity score^13^.

The aftermath of dietary modifications made during pregnancy has the most substantial effects on nutritional status and birth outcomes, despite the important influence of mothers’ nutritional reserves^14^. The dietary diversity of pregnant individuals can be determined using the minimum dietary diversity for women (MDD-W)^15^. Eating different foods may not always reflect a healthy diet. This is because a person may eat various items made from the same food group (e.g., cereals). As a result, the various food groups ingested by individuals are typically used as indicators of diet quality^16^.

A cross-sectional study conducted among pregnant individuals in Northeast Ethiopia revealed that the magnitude of inadequate dietary diversity practice was 69.6%^17^, whereas a study conducted in Eastern Ethiopia showed that 84.8% had suboptimal dietary diversity^18^; moreover, a study in Gindeberet revealed that 44.4% of pregnant individuals had inadequate dietary diversity^19^. Different causes were reported to be associated with inadequate dietary diversity practices, such as food taboos, poor nutritional literacy and pregnancy complications^20^, antenatal care, family size ≥ 5, the use of unprotected sources of water and rural residence^19^, maternal education and wealth indices^17^, and food insecurity^21^.

Inadequately diversified food consumption during pregnancy can lead to micronutrient deficiencies, including anemia, which can affect maternal and newborn outcomes^6,22^ and can also result in preterm delivery, low birth weight^22^, intrauterine growth restriction and abortion^19,23^. There have been promising nutritional interventions for adolescents in Ethiopia in the last two decades. Adolescent nutritional implementation guidelines were developed in 2023, and they focus on preventing the intergenerational cycle of malnutrition that occurs during adolescent pregnancy^24^. There is weekly iron folic acid supplementation (WIFAS) and nutritional education^25^; however, Ethiopia still has a long way to go to meet adolescent and youth health and nutritional needs, and there are still key problems such as teenage pregnancy, unplanned pregnancy and compromised nutritional status^26^.

An analytical cross-sectional study of pregnant adolescents visiting health facilities in neighboring Kenya revealed a significant strong positive relationship between the nutritional knowledge score, the dietary diversity score, and the number of meals consumed^27^. Similar research in Sudan indicated that superior habits in terms of food selection and dietary diversity scores were demonstrated by greater knowledge^28^. Numerous studies have been conducted on dietary practices and their determinants among pregnant women in Ethiopia^29–32^, but there is a gap in the evidence concerning these practices among pregnant adolescents. Pregnant adolescents continue to constitute an underserved population; therefore, this study sought to close this gap by examining dietary practices and associated factors among pregnant adolescents in the West Arsi Zone, Central Ethiopia.

## Methods and materials

### Study setting and design

A community-based cross-sectional study was conducted among pregnant adolescents in West Arsi, central Ethiopia. The zone is in Oromia, 250 km from Addis Ababa, the capital city of Ethiopia. The West Arsi Zone has 16 districts (13 rural areas and 3 towns). The population was estimated to be 2,929,894 in mid-2022. The zone has an Sq.km area of 12,556, with a climate of 45.5% highland, 39.6% medium, and 14.9% lowland. Agriculture was the dominant means of livelihood in Zone^33^. The study was conducted between February and March 2023.

### Source population and study population

All pregnant adolescents in the zone were the source population, whereas those in the selected kebeles were the study population. The study units were pregnant adolescents within the selected kebeles.

### Inclusion criteria

Gestational age prior to 16 weeks of pregnancy, as this information is the baseline of a cluster-randomized controlled community trial that was registered with Pan African Clinical Trials.gov at www.pactr.org, under the identification code PACTR202203696996305.

### Exclusion criteria

Pregnant adolescents who were diseased or unable to provide data at the time of data collection were excluded.

### Sample size determination and sampling technique

Considering a single population proportion formula, the sample size was determined with the assumption of a proportion (p) of good diet diversity practice among pregnant individuals in Southwest Ethiopia, which was 14.7%^29^. 95% confidence level (Z α/2) and 0.05 expected margin of error (d).

$$\begin{aligned} & {\text{n}} = \frac{{({\text{Z}}\alpha /{2})^{{2}} \cdot {\text{P}}\left( {{1} - {\text{P}}} \right)}}{{\left( {\text{d}} \right)^{{2}} }} = \frac{{({1}.{96}){ 2} \cdot \, 0.{15}\;({1}{-}0.{85})}}{{(0.05)^{2} .}} \\ & {\text{n}} = {3}.{84} \times \left( {0.{15} \times 0.{85}} \right)/0.00{25} \approx {196}. \\ \end{aligned}$$

Considering the design effect of 2 and adding a 10% nonresponse rate, the sample size was 431; however, the current study is part of follow up study [PACTR202203696996305.] registered at www.pactr.org, and sample size calculated for other objective was 488, which is greater than this, therefore sample size for this study was 488.

Pregnant adolescents were identified using a multistage clustered sampling technique. Four districts were chosen at random from 16 districts of the zone in the first stage. The second stage involved selecting kebeles, the lowest administrative entity, at random from the districts that had been chosen. After that, pregnant adolescents were counted during house-to-house visits to each of the chosen kebeles, and all pregnant adolescents visiting the selected kebeles were included in the study. The last menstrual period (LMP) was used to determine pregnancy among pregnant adolescents, and a pregnancy test was used to confirm pregnancy. The health extension workers and women’s development army served in the identification of pregnant adolescents. Six clusters were from both Dodola Rural Adaba districts, and eight clusters were from both Gedeb Hasasa and Siraro districts.

### Data collection procedure and measurements

The data were gathered using a pretested structured questionnaire. The questionnaire included questions on sociodemographic factors, meal frequency, health service usage, dietary categories, HFIAS (Household Food Insecurity Access Scale), crop diversity, and livestock diversification. Data on the HBM constructs were collected from two MPH (master of public health) holders and six clinical nurses who were hired as supervisors and data collectors, respectively. Three female laboratory technicians were also employed to perform pregnancy tests. At the participants' residences, the data collectors conducted face-to-face interviews to complete the questionnaire. Other family members were not allowed to access the location where the interviews took place to protect the pregnant adolescents’ privacy to the fullest extent possible.

Dietary intake was calculated using multiple-pass 24-h recalls in accordance with the Food and Nutrition Technical Assistance (FANTA) III recommendation from the Food and Agricultural Organization and the United States Agency for International Development. The goal was to determine whether pregnant teenagers' diets are diverse^34^. According to the recommendations, there are ten food groups, including grains, plantains, white roots and tuber pulses (peas, beans, and lentils), dairy plants, meat, nuts and seeds, eggs, dark green leafy vegetables, poultry and fish, other vegetables and other fruits, and other fruits and vegetables rich in vitamin A. A pregnant adolescent was deemed to be consuming a sufficiently diverse diet if she had consumed at least five of the foods on the aforementioned lists in the 24 h prior to the data collection period^35,36^. Participants were asked to recall every meal they had and drink they had consumed during the preceding 24 h, both inside and outside of the home. In addition, the participants were prompted to recall any between-meal snacks they may have had. Food items were scored as "1" for consumption over the reference period and "0" for nonconsumption.

The HFIAS Guideline^36^ was used to assess food security. Twenty-seven questions on the Household Food Insecurity Access Scale were used to evaluate the households’ level of food security. Prior to this, the questions were approved for use in underdeveloped nations^37^. Food-secure households experienced fewer food insecurity indicators than did the first two food insecurity indicators. Households that experienced between two and ten, 11–17, or more than 17 food insecurity indicators were considered mildly, moderately, or severely food insecure, respectively.

Using principal component analysis (PCA), the household's wealth index was calculated by considering access to a latrine, a water source, household possession, animals, and agricultural land. The responses of the nondummy variables were divided into three categories. The highest rating is assigned a code of 1. However, the two lower numbers received a code of 0. In PCA, factor scores were generated using variables with a commonality value greater than 0.5. To calculate the wealth score, the first primary component score for each family is retained. To categorize households as poorest, poor, medium, rich, or richest^31^, quintiles of the wealth score were developed^31^.

Eight questions were used to measure the autonomy of pregnant adolescents. When a choice was made by the woman alone or jointly with her husband, code one was assigned for each question; otherwise, code zero was assigned. The ability of pregnant adolescents to make decisions was categorized according to the mean score^38^.

With the help of 12 questions, maternal knowledge of food intake during pregnancy was evaluated. When a response was correct, a code of one was issued; otherwise, a code of zero was given. Twenty items on the Likert scale were used to analyze attitudes via PCA. The factor scores were summed and divided into three portions, or terciles. The highest tercile was then classified as favorable, if not unfavorable^31^.

### Operational definitions

Adequate dietary diversity: Pregnant adolescents who consumed food from 5 or more food groups within 24 h of day/before data collection [MDD-W, minimum dietary diversity for women was used]^34^.

Inadequate dietary diversity: Pregnant adolescents consumed food from fewer than 5 food groups on the day/within 24 h before data collection^34^.

Nutrition knowledge: Pregnant adolescents’ knowledge of optimal nutrition during pregnancy was scored as follows: Pregnant adolescents with scores ≥ 75 and > 50% from 12 knowledge questions were considered to have high and medium knowledge scores, respectively. Otherwise, they are considered to have a low knowledge score^39^.

### Data quality control

By employing a standard, adapted questionnaire and adhering to the steps required to produce the desired results, the highest possible level of data quality was ensured. To maintain the uniformity of the questions, the questionnaire was translated into the local languages Amharic and Afan Oromo and then translated back into English. Pretesting was performed using a 5% sample size. Based on the gaps found during the pretest interview, changes were made to the data collection methods to improve reliability. The principal investigator provided a two-day training session for the data collectors and supervisors on the goal and methodology of the data collection, and they also participated in mock interviews and actual field exercises. To ensure the accuracy of the data, supervisors monitored the field practices and reviewed the completed surveys each day.

#### Reliability and validity of the data collection tools

A test–retest method was used for reliability analysis of the pregnant adolescent nutritional knowledge variable, and questions were reviewed to assess whether the results were the same for all respondents. A Cronbach coefficient of 0.83 was obtained after the pretest. To ensure validity, nutrition experts from Dilla University, the Department of Nutrition, were involved in checking the questionnaire to ensure that the questions elicited the required response, and a pretest was also conducted.

### Data management and statistical analysis

The data were collected using Kobo Collect/Toolbox and subsequently exported to SPSS version 25 for cleaning and analysis. The intraclass correlation coefficient (ICC) was calculated and found to be 0.01, suggesting that there was little variation between groups and that the group-level effect was negligible; therefore, the traditional logistic regression model was used in this study. To identify independent predictors of dietary diversity, a binary logistic regression model was applied. In the bivariate analysis, variables with p values less than 0.25 were added to the multivariate logistic regression model. The 95% confidence intervals (CIs), crude and adjusted odd ratios (CORs) and AORs were used to calculate the strength of the relationship. According to the multivariate logistic regression analysis, variables with a P value of 0.05 or lower were regarded as independent predictors of dietary diversity. Collinearity between predictor variables was checked using the variance inflation factor (VIF); the maximum VIF was 2.8.

### Ethical approval and consent to participate

Ethical approval was obtained from the Jimma University IRB/ethics committees with reference number JUIH/IRB/194/22 and the Oromia Regional Health Office. All methods were performed in accordance with the relevant guidelines and regulations and in accordance with the Declaration of Helsinki. Informed consent was obtained from all participants and/or their legal guardians. Each study participant received a thorough description of the study's title, goal, protocol, and duration, as well as the potential risks and benefits, prior to providing informed consent. Each teenager provided verbal, written, and signed informed consent prior to any interview or measurement. Participants were made aware of the publication of their anonymous comments. Informed consent was obtained from participants prior to the commencement of interviews. The researcher remained truthful to the academic and ethical requirements. Finally, the researcher kept the data in a locked file cabinet in a safe place after the completion of the study. Informed consent was obtained from both the adolescent and their husband or parents. Any ethical issues that arose during this research were resolved through discussion between the researcher and JU’s IRB. Finally, nutritional education interventions based on the results were continued after this study.

## Results

### Sociodemographic and obstetric characteristics of pregnant adolescents

A total of 459 pregnant teenagers participated and response rate was 94%. The mean (± SD) age of the pregnant adolescents was 18.2 (± 0.97) years. According to the wealth index, only 17.4% of the respondents were classified as the richest people (Table 1).

**Table 1:** Sociodemographic and economic characteristics of pregnant adolescents in west Arsi zone, central Ethiopia, 2023 (n = 459).

Variable	Frequency	Percent
Age in years		
15–17	103	22.4
18–19	356	77.6
Educational status		
No formal education	10	2.1
Can read and read	23	4.9
Primary School	290	62.1
High school	96	20.6
College and above	40	8.6
Marital status		
Married	417	91
Single	37	8
Divorced	5	1
Family size		
< 4	235	51
≥ 4	224	49
Occupational		
House wife	233	50.7
Student	147	32
Merchant	60	13
Daily laborer	6	1.3
Government employer	13	2.8
Husband’s education		
No formal education	15	3.2
Can read and write	96	20.9
Primary school	251	54.6
High school	56	12.2
College and above	41	8.9
Husband’s occupation		
Merchant	88	19
Daily laborer	36	7.8
Government employer	96	20.9
Private employee	48	10.5
Farmer	191	41.6
Wealth index		
Poorest	90	19.6
Poor	108	23.5
Medium	50	10.9
Rich	131	28.5
Richest	80	17.4

### Nutrition knowledge and attitudes of the participants

The nutritional knowledge of the study participants was assessed; accordingly, 269 (57.6%) of the respondents had a low level of nutritional knowledge. Approximately 338 (72.4%) had unfavorable attitudes toward good nutritional practices during pregnancy.

### Obstetrics and health service-related characteristics

Approximately 122 (26%) of the pregnant adolescents underwent ANC follow-up, and a total of 170 (37%) of them received nutrition education during their follow-up (Table 2).

**Table 2:** Obstetrics and health service-related characteristics of pregnant adolescents in the West Arsi Zone, central Ethiopia, 2023 (n = 459).

Variable	Frequency	Percent
ANC follow up started		
Yes	122	26.1
No	337	73.9
Nutrition education		
Yes	170	36.4
No	289	61.9
Gestational age		
Less than 12 weeks	157	34.2
12–16 weeks	302	65.8
Gravida		
Primigravida	412	89.7
Multigravida	47	10.3
Nausea and vomiting		
Yes	392	85.4
No	67	14.6

### Inadequate dietary diversity of participants

**Figure 1:**
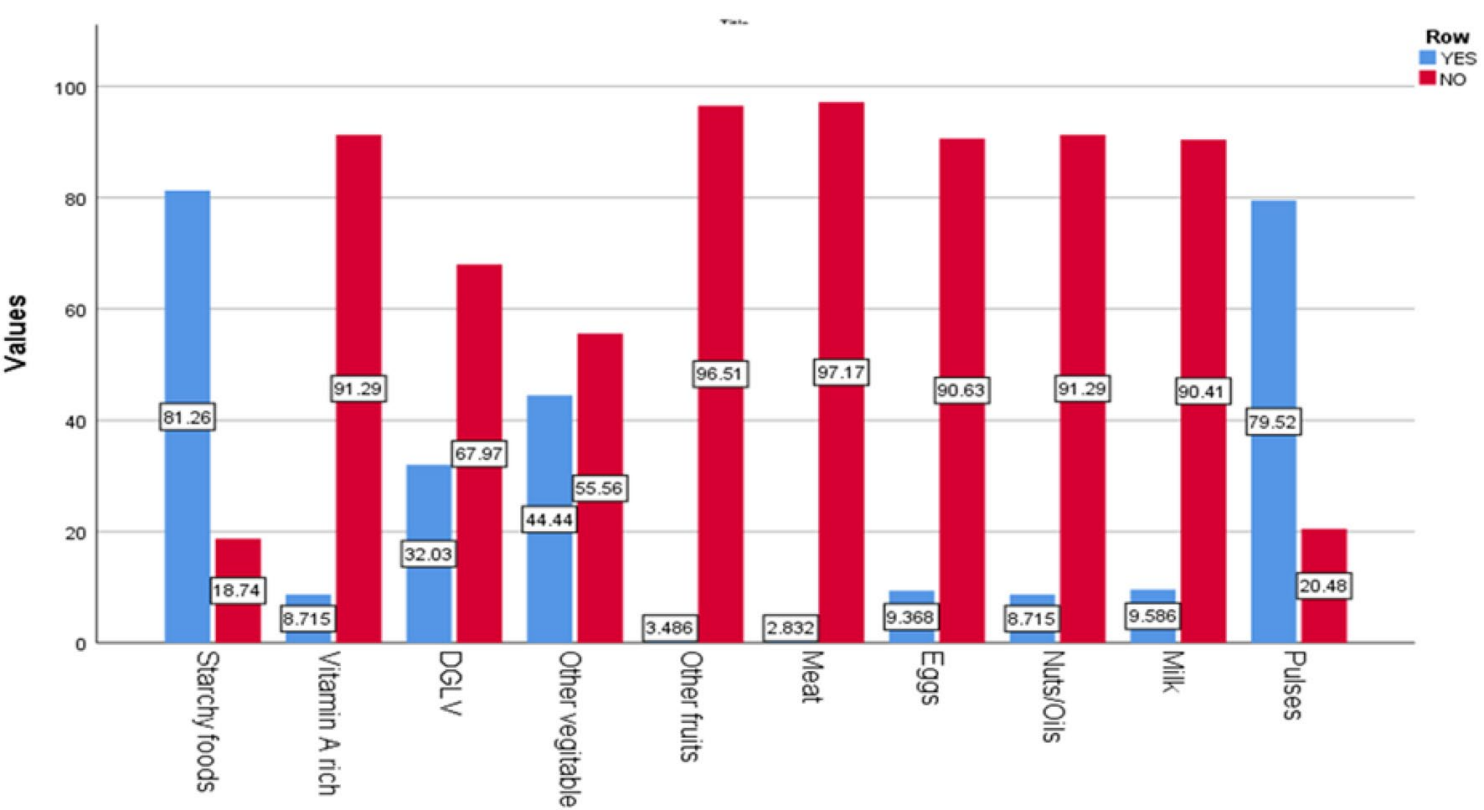
Dietary diversity practices of pregnant adolescents in the West Arsi zone, central Ethiopia.

Seventy-eight percent of the pregnant adolescents had inadequate dietary diversity practices during the preceding 24 h of the survey (95% CI 74.3%, 82.8%), and 21% had adequate dietary diversity practices. The most frequently eaten foods were starchy foods (81.3%) and pulses (79%), and the least consumed foods were meats (2.8%) and fruits (3.48%) (Fig. 1).

### Factors associated with inadequate dietary diversity practices

Nutritional knowledge, attitudes toward acceptable dietary practices, food security, perceived susceptibility, household decision making, receiving nutritional education, perceived benefits and perceived severity were associated with dietary diversity practices according to bivariate logistic regression analysis. Multivariate logistic regression analysis revealed that nutritional knowledge, attitudes toward acceptable dietary practices, food security and perceived severity of poor dietary practices were significantly associated with the dietary practices of pregnant adolescents.

The odds of having inadequate dietary practices among pregnant adolescents who had a low level of nutritional knowledge were approximately two times greater than those who had a high level of nutritional knowledge [AOR = 2.4, 95% CI 1.82–4.74]. Regarding attitudes, the odds of having inadequate dietary practices among pregnant adolescents who had unfavorable attitudes toward acceptable dietary practices were four times greater than those who had favorable attitudes [AOR = 4.3, 95% CI 2.9–5.83]. The odds of having inadequate dietary practices among food insecure pregnant adolescents were approximately nine times greater than those among food secure adolescents [AOR = 8.7, 95% CI 7.37–10.24]; additionally, the odds of having inadequate dietary practices among pregnant adolescents who had low perceived severity of poor dietary practices were four times greater than those who had high perceived severity [AOR = 4.7, 95% CI 3.26–5.47] (Table 3).

**Table 3:** Factors associated with inadequate dietary diversity score among pregnant adolescents in the west Arsi zone, central Ethiopia, 2023 (n = 459).

Variables	Dietary diversity practice		COR (95% CI)	AOR (95%CI)
	Inadequate	Adequate		
Knowledge				
Low	241	28	2.75(1.3–7.64)	2.4(1.82–4.74) **
Medium	94	63	0.47(0.2–0.87)	0.32(0.29–0.43)
High	25	8	1	1
Attitude				
Unfavorable	315	23	23(18.4–26.74)	4.3 (2.9–5.8) ***
Favorable	45	76	1	1
HH Decision making				
No	309	19	25.5(16.74–29.83)	3(1.95–6.2)
Yes	51	80	1	1
Food security				
No	312	25	19.24(11.47–21.8)	8.7(7.37–10.24) ***
Yes	48	74	1	1
Nutrition education				
No	280	9	35(32.4–54.55)	8.4(7.2–10.6)
Yes	80	90	1	1
Perceived severity				
No	306	36	9.9(4.37–16.47)	4.7(3.26–5.47)**
Yes	54	63	1	
Perceived benefits				
No	313	21	24.7(11–27.38)	3.7(2.33–5.96)
Yes	47	78	1	

*Significant at P ≤ 0.05, **significant at P ≤ 0.002, ***significant at P ≤ 0.001, OR = odds ratio, AOR = adjusted odd ratio, CI = confidence interval.

## Discussion

This study aimed to assess dietary diversity practices and associated factors among pregnant adolescents. Seventy-eight percent of the pregnant adolescents had inadequate dietary diversity practices, and nutritional knowledge, attitudes toward acceptable dietary practices, food security and perceived severity of poor dietary practices were associated with the dietary practices of pregnant adolescents.

The Food and Agriculture Organization (FAO) and World Health Organization (WHO) recommend that all mothers attain minimum dietary diversity scores to ensure micronutrient adequacy^34^. The magnitude of inadequate dietary diversity in this study was greater than that reported in several related studies, such as those conducted in Ghana (56%)^40^, South Africa (75%)^41^, Kenya (39%)^42^, the Gurage Zone, Southwest Ethiopia (58%)^43^ and the Jimma Zone, Southwest Ethiopia (48.3%)^44^. Possible explanations for this might be that because the study was conducted among pregnant adolescents and because the aforementioned studies, except for the study conducted in Ghana, involved pregnant adults. This might be caused by differences in food security, employment level, and nutritional knowledge. Moreover, pregnant adolescents with less than 16 weeks of gestation were included, and the majority were primigravida, which had the highest incidence of hyperemesis gravida; these conditions may alter dietary practices and lead to food selection. Due to their low dietary diversity, pregnant teenagers are more likely to be deficient in nutrients, particularly micronutrients, which could affect the chances of having a healthy pregnancy outcome.

The other main finding of this study was that the odds of having inadequate dietary practices among pregnant adolescents who had a low level of nutritional knowledge were greater than those among pregnant adolescents who had a high level of nutritional knowledge, and these findings are consistent with those of studies conducted in Khartoum, Keniya^45^, Ghana^46^, West Kenya^47^ and China^48^. This could be explained by the fact that changes in attitudes and knowledge are important antecedents of behavioral change, and nutritional knowledge is predictive of changes in dietary habits^49^.

Dietary practices can improve during pregnancy because pregnant individuals are more interested in learning about food and nutrition than they are before conception^50,51^. Additionally, different studies conducted in Ethiopia have shown that knowledge of nutrition among pregnant individuals is lower^52,53^; therefore, nutritional behavioral change communication interventions (NBCCs) should be designed and delivered well for pregnant individuals, especially pregnant adolescents who are immature, at higher risk, hard to reach and underserved. The NBCC needs to be based on behavior change models and focus on the benefits of a diversified diet.

Adolescents frequently become pregnant unintentionally, and studies in Ethiopia revealed that only approximately 27% of them receive antenatal care (ANC) services^54,55^. As a result, they miss out on crucial nutritional education and adopt unhealthy eating habits, which makes them even more susceptible to undernutrition and other negative health outcomes. The government should oversee its implementation and enhance the quality of nutrition education because it is a less expensive and more efficient technique, particularly in areas with limited resources.

The odds of having inadequate dietary practices among pregnant adolescents who had unfavorable attitudes toward acceptable dietary practices were greater than those participants with favorable attitudes. This evidence is supported by studies conducted in southern Ethiopia^56^, the Gojjam zone, northern Ethiopia^31^, and Kigeme, Rwanda^57^. A possible explanation for this difference might be that pregnant adolescents with a positive attitude toward their maternal diet are more likely to intend to consume a balanced diet, which has a direct impact on their dietary practices.

Another finding is that the odds of having inadequate dietary practices among food insecure pregnant adolescents were greater than those among food secure adolescents. This result is in agreement with the findings of studies conducted in the Illu Aba Bor Zone, southwestern Ethiopia^32^, Tigray, Ethiopia^58^ and the Gurage Zone, southern Ethiopia^43^. Adolescents are exposed to coping mechanisms that force them to limit the types and amounts of food they consume when food supplies are scarce. In developing nations, nutrition policies, strategies, and programs need to pay more attention to adolescent nutrition in general and pregnant adolescent nutrition and food security in particular.

This study revealed that the odds of having inadequate dietary practices were greater among pregnant adolescents who reported poor dietary practices than among those who reported low perceived severity. There are similarities between this evidence and those of studies conducted in northeastern Ethiopia^30^ and in the West, Gojjam Zone, and Ethiopia^59^. This may be because adolescents who were pregnant perceived that eating poorly during pregnancy had serious negative consequences for both them and their unborn child. Depending on how significant they perceive the consequences to be, a person's propensity to alter their health-related behavior is affected^60^.

One of the risk factors for healthy eating is the perceived severity of the effects of poor maternal diet, and it is one of the constructs of the health belief model (HBM). An interpersonal model of health called the HBM is used to promote constructive behaviors^61^. It clarifies why some people make efforts to improve their health while others do not. Its constructs consist of perceived susceptibility, severity, barriers to a specific behavior, benefits, self-efficacy and cues to action^61,62^. In nutrition education sessions, health care providers should consider using models such as the HBM. It is important to explain and discuss during counseling the negative effects of eating inadequate amounts of unbalanced meals, the vulnerability to and severity of those negative effects, the advantages of eating a healthy diet, and the obstacles to eating a balanced diet.

## Strength and limitations

In this study, individuals consumed meals within the previous 24 h. are taken into account, which may not accurately reflect their typical eating patterns. Dietary intake can change during bumper or lean seasons as well as during celebrations, none of which were taken into account in this study. The 24-h. The data do not represent the long-term dietary habits of the participants. Despite its limitations, this study has contributed new knowledge to the scientific world, particularly in the setting of Ethiopia, on the variables that affect dietary diversity among pregnant teenagers.

## Conclusion

Compared to the findings of other studies, this study showed that the extent of inadequate dietary practices was high. The study demonstrated that inadequate dietary practices in pregnant adolescents were associated with inadequate nutritional knowledge, an unfavorable attitude toward acceptable dietary practices, food insecurity, and the perceived severity of inadequate dietary practices. Pregnant adolescents should receive nutritional intervention that focuses on nutritional education based on behavioral modification models to enhance their dietary practices. Interventional studies to enhance dietary diversity practices are needed. Moreover, sustainable income-generating activities and strategies are recommended.

## Data Availability

The datasets used and/or analyzed during the current study are available from the corresponding author upon reasonable request.
